# Generation and characterization of genome-modified chondrocyte-like cells from the zebra finch cell line immortalized by *c-MYC* expression

**DOI:** 10.1186/s12983-022-00464-x

**Published:** 2022-06-11

**Authors:** Kyung Min Jung, Young Min Kim, Eunhui Yoo, Jae Yong Han

**Affiliations:** Department of Agricultural Biotechnology and Research Institute of Agriculture and Life Sciences, College of Agriculture and Life Sciences, Seoul National University, 1 Gwanak-ro, Gwanak-gu, Seoul, 08826 Korea

**Keywords:** Zebra finch, Immortalized cell line, *c-MYC*, Chondrocyte-like cells, Gene editing

## Abstract

**Background:**

Due to their cost effectiveness, ease of use, and unlimited supply, immortalized cell lines are used in place of primary cells for a wide range of research purposes, including gene function studies, CRISPR-based gene editing, drug metabolism tests, and vaccine or therapeutic protein production. Although immortalized cell lines have been established for a range of animal species, there is still a need to develop such cell lines for wild species. The zebra finch, which is used widely as a model species to study the neurobiological basis of human speech disorders, has been employed in several functional studies involving gene knockdown or the introduction of exogenous transgenes in vivo; however, the lack of an immortalized zebra finch cell line has hampered precise genome editing studies.

**Results:**

Here, we established an immortalized cell line by a single genetic event, expression of the *c-MYC* oncogene, in zebra finch embryonic fibroblasts and examined its potential suitability for gene targeting investigations. Retroviral vector-mediated transduction of *c-MYC* was used to immortalize zebra finch primary fibroblasts; the transformed cells proliferated stably over several passages, resulting in the expression of chondrocyte-specific genes. The transfection efficiency of the immortalized cells was much higher than that of the primary cells. Targeted knockout of the *SOX9* gene, which plays a role in the differentiation of mesenchymal progenitor cells into chondrocytes, was conducted in vitro and both apoptosis and decreased expression levels of chondrogenic marker genes were observed in edited cells.

**Conclusions:**

The *c-MYC* induced immortalized chondrocyte-like cell line described here broadens the available options for establishing zebra finch cell lines, paves the way for in-depth biological researches, and provides convenient approaches for biotechnology studies, particularly genomic modification research.

**Supplementary Information:**

The online version contains supplementary material available at 10.1186/s12983-022-00464-x.

## Background

Cellular senescence and stress caused by cell culture precludes the indefinite culture of primary cells in vitro [1]. Immortalized cell lines do not require extraction from live animals and can provide relatively consistent and reproducible results compared to primary cells [2]. Consequently, immortalized cell lines have become an important tool for various applications, including analyses of drug efficacy and cytotoxicity, production of biopharmaceuticals or vaccines, and studies of gene function and genome editing [3]. Various immortalized cell lines have been established for numerous species [1] and modified cell lines with enhanced properties, such as improved transfection efficiency or protein yields, are being developed and used extensively [4, 5]. Several avian cell lines, particularly those from chickens, have been developed and commercialized; these cell lines have been immortalized using various methods, such as serial passaging, carcinogenic chemical treatment, and the introduction of oncogenes [6–9]. Given that numerous avian species are being used as experimental model animals, the number of endangered bird species is increasing, and cell resources are still limited, it is necessary to expand cell line development research into more diverse avian species.

The zebra finch (*Taeniopygia guttata*) is used as an experimental model animal for neurobiological research. In particular, zebra finches are useful for studies examining song development and auditory processing because they are able to learn vocalizations by imitating a singing adult, similar to humans who acquire spoken language [10]. Functional gene analyses of zebra finches are typically performed by transient knockdown or overexpression of genes of interest via in vivo electroporation or surgical injection of viruses into specific brain regions, both of which require a high level of technical skill [11–14]. Although some groups have generated transgenic zebra finches expressing a mutant foreign gene introduced by a viral system, precise genome-edited zebra finches have not yet been developed [15, 16]. In general, cell lines are used to analyze gene function and pre-validate the CRISPR editing system in vitro, but the zebra finch cell line is still lacking, and primary cells display rapid senescence and a low transfection efficiency [17]. Accordingly, a cell line with myogenic identity was generated recently by introducing SV40 large and small T antigens into zebra finch embryonic fibroblasts (ZEFs), and the utility of this cell line was demonstrated using the CRISPR/Cas9 knockout system [18].

Various methods of generating cell lines have been developed; the selection of an appropriate approach requires consideration of efficiency, applicability, and expected cell characteristics. Recently, several studies have demonstrated that primary cells from mammals can be immortalized by introducing a single reprogramming factor, *c-MYC* [19–21]. In 2007, the ability of *c-MYC* to immortalize human cells was demonstrated by its introduction into human primary foreskin fibroblasts via a retroviral system [20]. Subsequently, this method has been used to produce an immortalized mouse Kupffer cell line, providing a useful means to study the function of these cells in vitro [19]. In addition, porcine fibroblasts have been successfully immortalized by *c-MYC* expression and showed chondrocyte-like cell (CLC) characteristics [21].

In this study, we used *c-MYC* expression to establish an immortalized zebra finch cell line exhibiting CLC characteristics and verified its use for targeted gene editing and gene function studies. This approach broadens the available options for establishing zebra finch cell lines, paves the way for in-depth biological studies, and provides a convenient approach to genomic modification studies.

## Results

### The zebra finch immortalized cell line avoided cellular senescence and exhibited telomerase activity

**Fig. 1:**
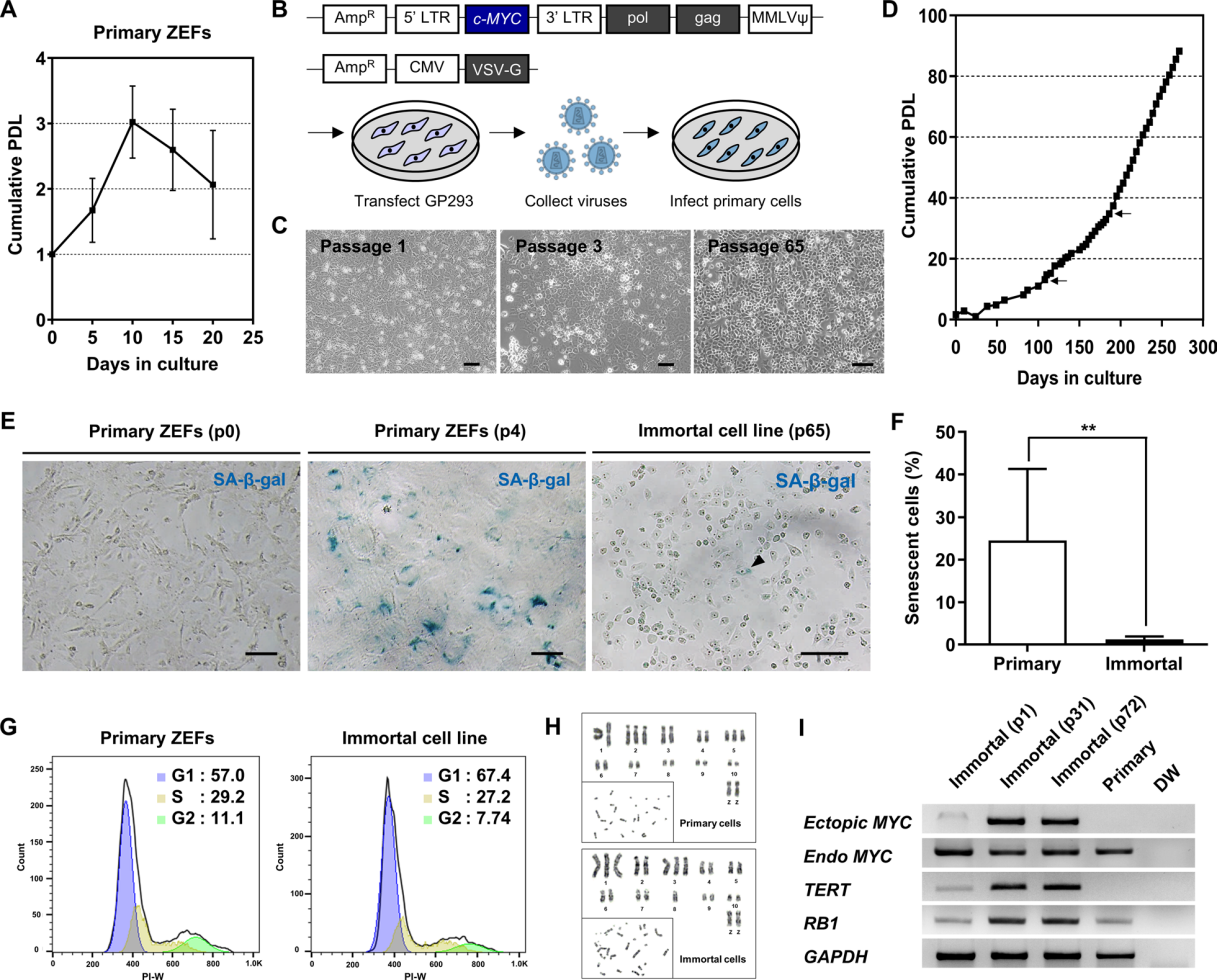
Immortalization of primary zebra finch embryonic fibroblasts (ZEFs) by *c-MYC* expression. **A** The growth curve of primary ZEFs. Subculturing was performed on days 5, 10, 15, and 20. PDL, population doubling level. **B** Schematic illustration of the immortalization of primary ZEFs via retroviral vector-mediated transduction. **C** The morphology of *c-MYC*-treated cells. Scale bar, 100 μm. **D** The growth curve of *c-MYC*-treated cells. **E** Cellular senescence of primary cells and the immortalized cell line. The black arrowhead indicates a stained (scenescent) cell. Scale bar, 100 μm. SA‐β‐Gal, senescence‐associated-β-galactosidase. **F** The percentages of senescent primary and immortalized cells; **p < 0.01. **G** Cell cycle analyses of primary cells and the immortalized cell line. **H** Karyotyping analysis of primary cells at passage 1 and immortalized cells at passage 65. Karyotyping analysis only involved in the macrochromosomes of zebra finch. **I** Telomerase activity of the immortalized cell line at passages 1, 31, and 72. The gene expression levels of ectopic mouse *c-MYC* from the pMXs-*c-MYC* plasmid and endogenous zebra finch *c-MYC*, *TERT*, and *RB1* were analyzed by RT-PCR. DW, distilled water

To investigate the proliferative ability of primary ZEFs, we assessed the population doubling level (PDL) and found that it decreased sharply after passage 2 (Fig. 1A). To generate an immortalized cell line, primary ZEFs at early passage numbers were transduced with retroviruses carrying the mouse *c-MYC* gene (Fig. 1B). Transduced cells underwent a morphological change at passage 3 (Fig. 1C), successfully bypassed cellular senescence, and continued to divide for more than 250 days (Fig. 1D). Only 1.24% of the immortalized cells displayed senescence at passage 65, whereas 24.5% of the primary ZEFs displayed senescence at passage 4 (Fig. 1E, F). Cell cycle analyses revealed similar profiles for the immortalized cells and the early passage primary ZEFs (Fig. 1G), and a karyotyping analysis revealed that both primary and immortalized cells showed aneuploidy on two or three different chromosomes (Fig. 1H and Additional file 1: Fig. S1). The number of karyotyped cells for each line was 20, and all karyotyped cells displayed the same karyotype. The telomerase activity of the immortalized cell line was confirmed by *TERT* and *RB1* gene expression levels, which became more robust with passage of the cell line (Fig. 1I).

### The zebra finch immortalized cell line showed chondrocyte-like characteristics

**Fig. 2:**
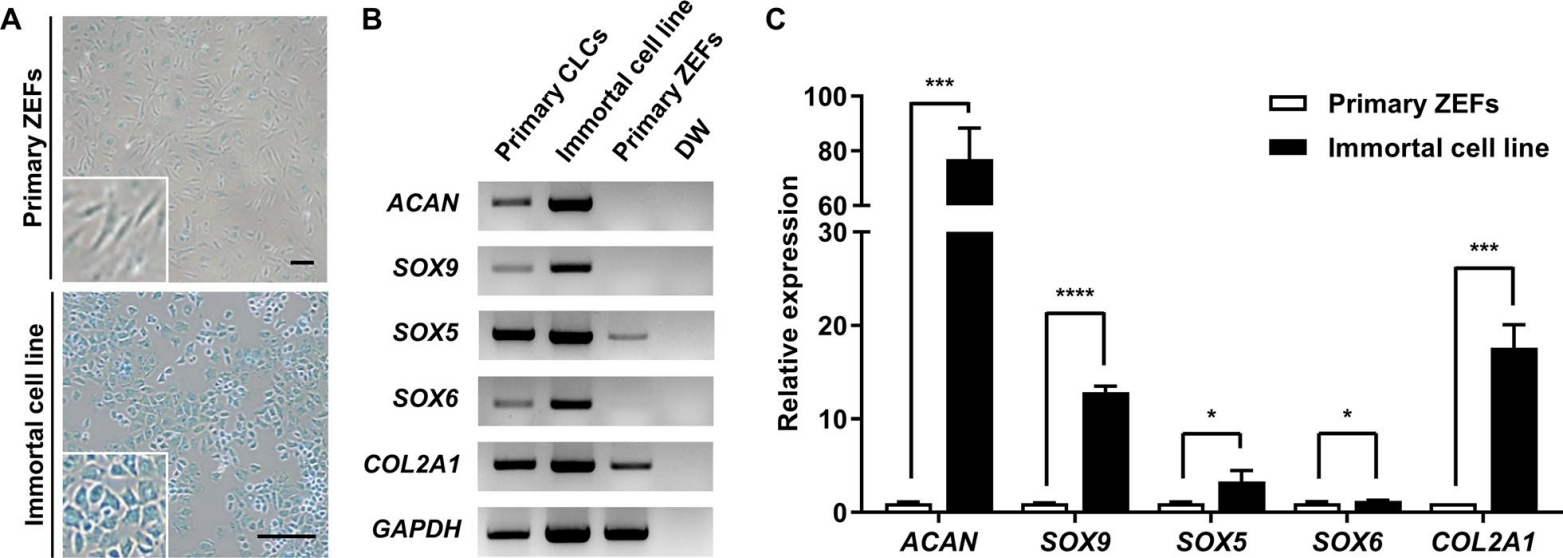
Characterization of the immortalized zebra finch cell line. **A** Alcian blue staining of primary zebra finch embryonic fibroblasts (ZEFs) and the immortalized cell line. Scale bar, 100 μm. **B** Gene expression profiling of the primary ZEFs, the immortalized zebra finch cell line, and primary chondrocyte-like cells (CLCs). DW, distilled water. **C** Quantitative RT-PCR analyses of the primary and immortalized cells; *p < 0.05, ***p < 0.0005, ****p < 0.0001

The immortalized cells displayed chondrocyte-like morphology and were stained intensely with Alcian blue (Fig. 2A). In addition, the immortalized cells expressed higher levels of chondrocyte marker genes, including *ACAN*, *SOX5*, *SOX6*, *SOX9*, and *COL2A1*, than the primary ZEFs (Fig. 2B, C), supporting the proposal that they had developed into CLCs.

### The immortalized cell line displayed enhanced transfection efficiency

**Fig. 3:**
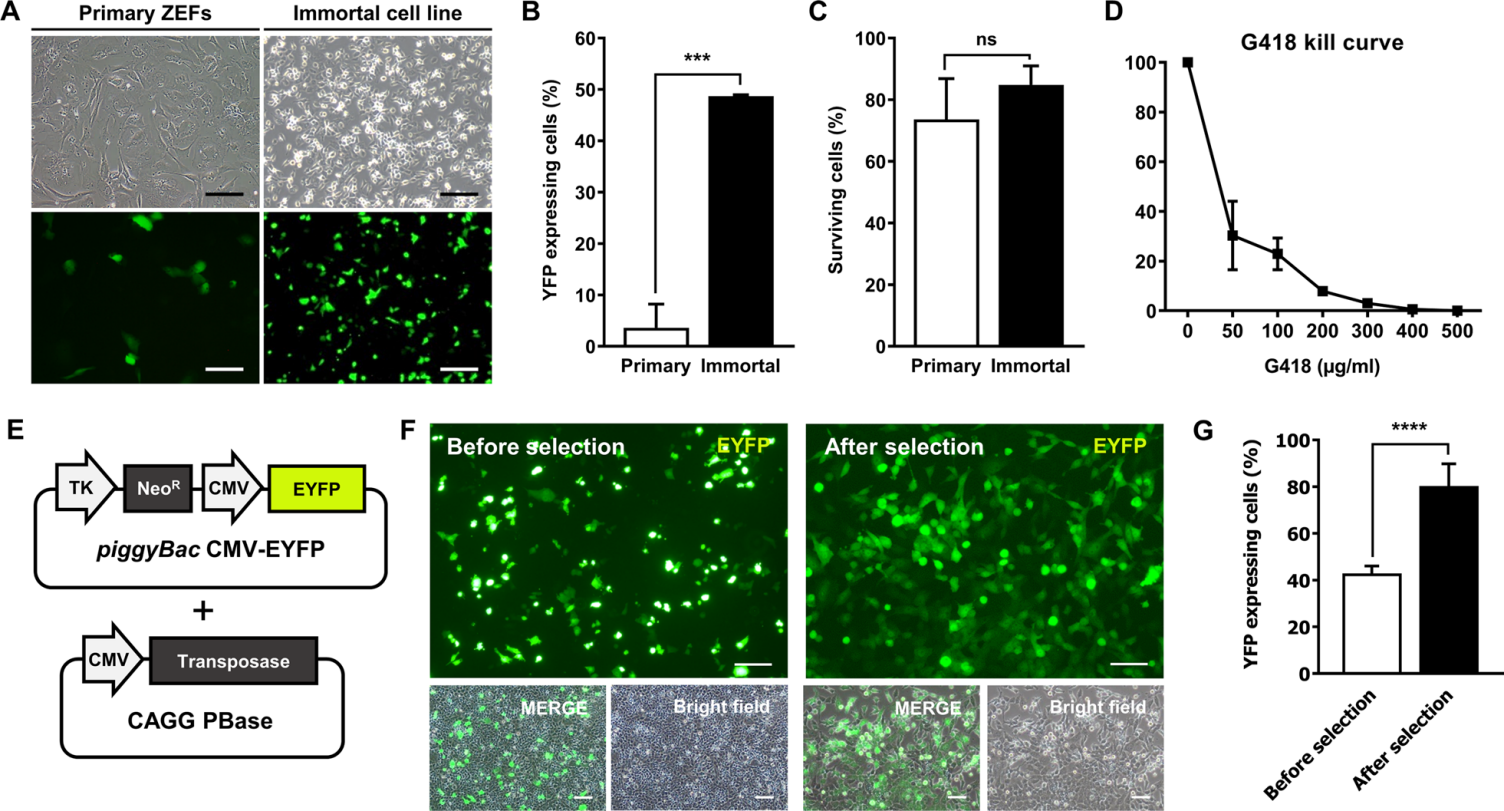
Enhanced transfection efficieny of the chondrocyte-like immortalized zebra finch cell line. **A** Representative images of transfected immortalized cells and primary zebra finch embryonic fibroblasts (ZEFs). Scale bar, 100 μm. **B** Transfection efficiencies of the primary and immortalized cells, as determined by flow cytometry. **C** The viabilities of the transfected primary and immortalized cells, determined via manual counting after trypan blue staining; ***p < 0.0001. **D** The sensitivity of chondrocyte-like zebra finch immortalized cells to G418. The cells were exposed to increasing concentrations of G418 for 1 week and the percentage survival was determined by trypan blue staining. **E** Schematic overview of the *piggyBac* YFP expression plasmid and the CAGG PBase plasmid. **F** Images of YFP-expressing immortalized cells before and after selection with G418 (300 μg/mL). Scale bar, 100 μm. **G** The percentages of YFP-expressing immortalized cells before and after G418 selection; ****p < 0.0001

To investigate the transfection efficiency of the immortalized cell line, primary ZEFs and immortalized cells were transfected with a YFP-expressing vector via lipofection (Fig. 3A). The immortalized cell line displayed a significantly higher proportion of YFP-positive cells (48.67 ± 0.31%) than the primary ZEFs (3.60 ± 4.60%) (Fig. 3B), whereas the viability rates of the two cell types were similar (84.83 ± 6.17% and 73.59 ± 13.25%, respectively) (Fig. 3C).

A G418 kill curve assessment was performed to determine the optimal concentration required to establish a stable transfected cell line. The lowest concentration of G418 that killed all non-transfected immortalized cells after a 1 week exposure period was 300 μg/mL (Fig. 3D). Subsequently, transfected immortalized cells were enriched by selection with 300 μg/mL G418 (Fig. 3E–G). These findings indicate that the transfection efficiency of immortalized cell lines is enhanced in vitro and these cells have the potential to be utilized for gene editing experiments.

### Targeted *SOX9* knockout in immortalized chondrocyte-like cells

**Fig. 4:**
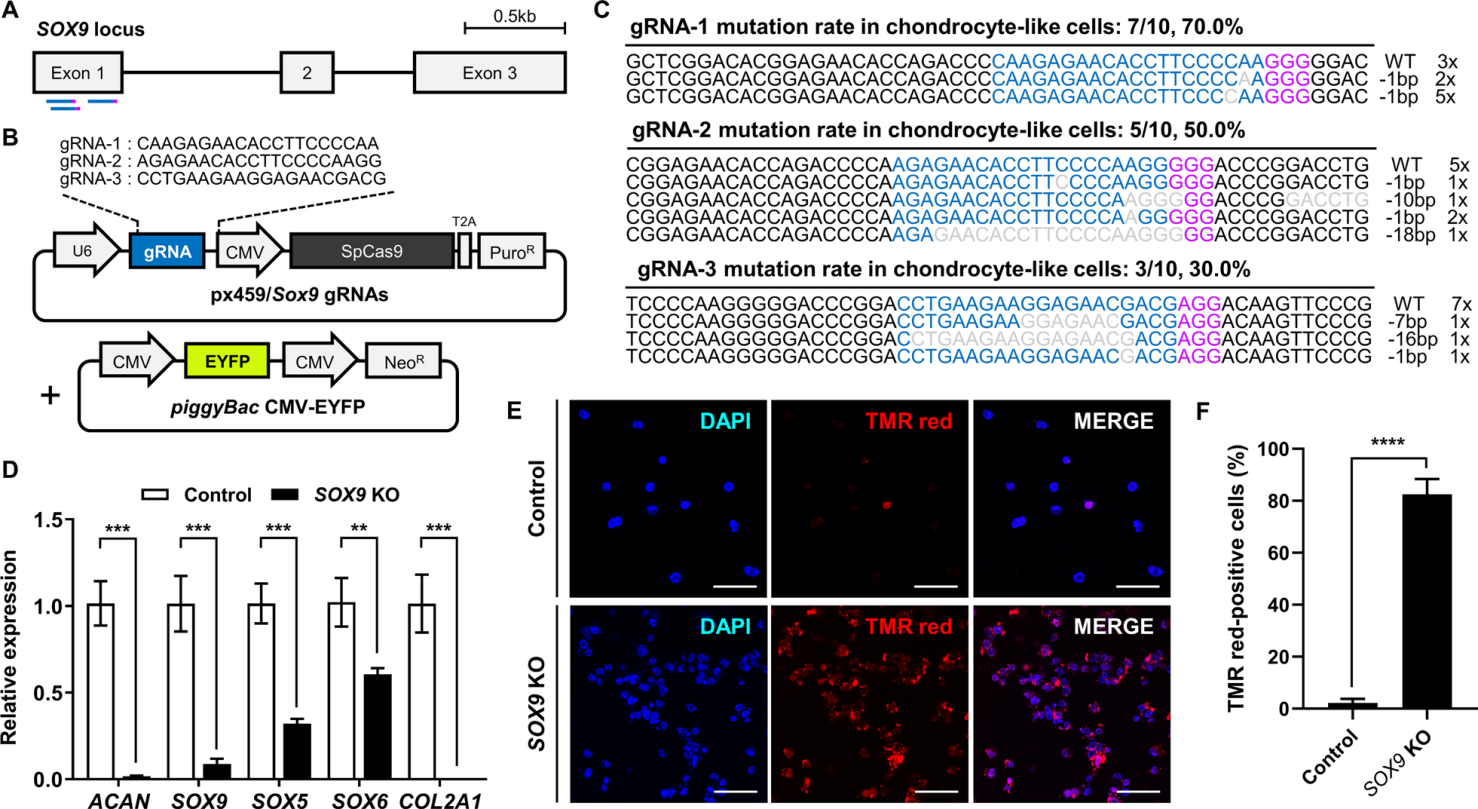
Targeted knockout of the *SOX9* gene in zebra finch chondrocyte-like cells (CLCs). **A** Schematic illustration showing the positions of the gRNAs used to target the zebra finch *SOX9* gene. **B** Sequences of the gRNAs and structure of the CRISPR/Cas9 plasmid targeting the *SOX9* gene. **C** DNA sequences of the wild type and mutated *SOX9* loci in transfected cells. Blue letters indicate gRNA recognition sequences, purple letters indicate PAM sequences, and gray letters indicate deletions. **D** The expression levels of chondrocyte marker genes in control (wild type) and *SOX9* knockout CLCs; **p < 0.01, ***p < 0.001. **E**, **F** Detection of apoptotic control and *SOX9* knockout CLCs. The cells were treated with the TUNEL reaction mixture (TMR red labeling) and apoptotic cells were counted manually. Scale bar, 50 μm; ****p < 0.0001

The *SOX9* gene plays an important role in the differentiation of mesenchymal progenitor cells into chondrocytes, and its deficiency causes apoptosis in chondrocytes [22]. Thus, we selected *SOX9* (located on chromosome 18) as the target gene to verify the gene editing potential of the zebra finch immortalized CLCs. Cells were transfected with CRISPR/Cas9 plasmids encoding specific gRNAs targeting exon 1 of the *SOX9* gene (Fig. 4A, B). To enable optimized G418 selection, the cells were also co-transfected with a *piggyBac* plasmid containing a neomycin resistance gene (Fig. 4B). Genomic DNA sequencing analysis confirmed that gRNA sequences 1, 2, and 3 induced nucleotide deletions at the targeted locus in immortalized zebra finch CLCs with efficiencies of 70%, 50%, and 30%, respectively (Fig. 4C). Therefore, we used zebra finch CLCs edited with gRNA-1 for subsequent experiments.

Quantitative RT-PCR analyses of control (wild type) and *SOX9 *knockout immortalized zebra finch CLCs revealed decreased expression levels of most chondrocyte-related genes in the knockout cells, including *ACAN*, *SOX5*, *SOX6*, and *COL2A1*, as well as *SOX9* (Fig. 4D). This result indicates that *SOX9* deficiency affects the expression of interacting genes in zebra finch CLCs. Furthermore, a TUNEL assay revealed that a high percentage of *SOX9* knockout CLCs were apoptotic (82.54 ± 5.86%), whereas only a few wild-type CLCs were apoptotic (2.24 ± 1.53%) (Fig. 4E, F), indicating that *SOX9* is required for the survival of chondrocyte lineage cells in zebra finch, as described previously for other species. These results demonstrate that immortalized zebra finch CLCs have the potential to be used for effective targeted gene editing and gene function analyses.

## Discussion

Immortalized cell lines have several advantages over primary cells but are only abundantly developed for major laboratory animals and are still species-limited. Primary cells can be used as an alternative, but senescence occurs quickly in vitro and the transfection efficiency of these cells is low [23, 24]. In addition, the senescence time point during in vitro culture of primary cells is different for each species [3]; in our current study, senescence of primary cultured ZEFs occurred very early (Fig. 1A), making it difficult to utilize these primary cells for research purposes. Therefore, we established an immortalized zebra finch cell line and characterized its usefulness.

A previous study generated immortalized cells with myoblast characteristics by introducing SV40 T antigen into ZEFs [18]. To expand the approaches for establishing immortalized cell lines in zebra finch, we introduced *c-MYC* into primary cells and generated a stably proliferating immortalized cell line displaying the characteristics of chondrocytes. The proto-oncogene *c-MYC* plays important roles in modulating cell cycle progression, proliferation, growth, self-renewal, differentiation, and apoptosis [21, 25]. In addition, *c-MYC* can extend the lifespan of fibroblasts and induce complete immortalization of normal human epithelial cells by activating telomerase [26]. The immortalized cell line described here went through a selection step, as shown by the inflection point of the growth curve (Fig. 1D), which might be considered a result of increased *c-MYC* expression, as seen for the human *c-MYC*-immortalized fibroblast cell line [20]. In addition, aneuploidy was observed in both the primary zebra finch cells and the immortalized cell line (chromosome 2, 5 in primary cells; chromosome 1, 3, 8 in immortal cells). Zebra finch *c-MYC* is located on chromosome 2, suggesting that overexpression of exogenous *c-MYC* is not directly related to the abnormal karyotype. Natural chromosomal abnormalities occur at a rate of approximately 5% in zebra finch, and these abnormalities cause mortality during embryo development and affect the hatching rate [27]. And the additional karyotyping of several primary cells revealed various chromosomal abnormalities (Additional file 1: Fig. S1). Therefore, we propose that, in the future, karyotyping should be prioritized when establishing a cell line derived from embryonic zebra finch cells.

The immortalized zebra finch cell line developed here showed chondrocyte-like characteristics. In previous studies, mouse and human dermal fibroblasts were transformed into CLCs by the introduction of defined factors such as the reprogramming factors *c-MYC* and *KLF4* and the chondrogenic factor *SOX9* [28, 29]. In addition, porcine CLCs were generated by transduction of *c-MYC* only into porcine embryonic fibroblasts [21]. In our current study, the embryonic tissues sampled for primary culture of fibroblasts consisted of a mixed population of several morphologically and functionally heterogeneous cells; thus the heterogeneity of the primary cells may have influenced the characteristics of the immortalized cell line [8, 30].

The electroporation-mediated transfection efficiency of primary ZEFs has been reported previously as 40%, with a cell viability of less than 50%, and the gene editing efficiency has been reported as up to 30% [31]. The immortalized cell line described here showed an improved lipofection-based transfection efficiency of approximately 50%, with high cell viability. Since immortalized cell lines can be cultured indefinitely, the transfection efficiency could be increased further by antibiotic selection (Fig. 4). Consequently, we were able to target knockout of the *SOX9* gene and thus confirmed the potential application of the immortalized cell line to study cell characteristics and related genes. In addition to its ability to establish stable genetically modified cell lines, the immortalized zebra finch cell line described here could also be used to validate precision genome editing systems such as targeted knock-in or base editing.

In conclusion, this study demonstrates that primary fibroblasts derived from zebra finch embryos can be successfully immortalized by retroviral transduction of *c-MYC* alone; these cells can undergo stable proliferation and achieve higher transfection efficiencies than primary cells. These characteristics support effective gene editing in vitro and will enable the use of the cells for future gene function studies or validation of CRISPR systems. We expect that the approach described here can also be applied to avian species other than zebra finch.

## Methods

### Experimental animals

All methods were performed in accordance with the ARRIVE (Animal Research: Reporting of In Vivo Experiments) guidelines and were approved by the Institutional Animal Care and Use Committee (IACUC, SNU-210217–5) of Seoul National University.

### Primary culture of zebra finch fibroblasts and chondrocyte-like cells

Primary ZEFs were prepared from 6-day-old embryos. Collected tissues were dissociated with 0.05% trypsin–EDTA and resuspended in Dulbecco’s Modified Eagle Medium (Hyclone, Logan, UT, USA) containing 10% fetal bovine serum (Hyclone) and 1 × antibiotic–antimycotic (Thermo Fisher Scientific, Waltham, MA, USA). Cells were seeded at a density of 1 × 10^6^ cells per 100 mm dish. When grown to confluent layers, cells were considered to be at population doubling level (PDL) zero and were passaged to calculate the PDL. The PDL test was carried out as described previously [3]. Primary CLCs were cultured as described previously and were used as a positive control in RT-PCR analyses [32].

### Retroviral infection and culture of immortalized cells

Retroviral vector particles were produced by the calcium phosphate co-precipitation method, as described previously [33]. Briefly, the retroviral pMXs-*c-MYC* vector (Addgene, Cambridge, MA, USA) containing the mouse *c-MYC* gene was introduced into GP293 cells along with the pVSV-G packaging plasmid (Invitrogen, Thermo Fisher Scientific Inc., Carlsbad, CA, USA). Target cells were exposed to retroviruses for 48 h. Transduced cells were maintained in the same growth medium as the primary cells. When grown to confluent layers, cells were considered to be at PDL zero and were passaged to calculate the cumulative PDL.

### Senescence analysis

Primary ZEFs and immortalized cell lines were fixed and then stained using the Senescence β-Galactosidase Staining Kit (Cell Signaling Technology, Danvers, MA, USA).

### Cell cycle analysis

Cells were treated with 10 μg/mL RNase A (Invitrogen) for 30 min at 37 °C and 50 μg/mL propidium iodide (Millipore Sigma, Burlington, MA, USA) for 30 min at 4 °C. The cell cycle status was determined using the FACSCalibur system (BD Biosciences, San Jose, CA, USA) and data were analyzed using FlowJo software (Tree Star, Ashland, OR, USA).

### Karyotype analysis

Primary ZEFs and immortalized cell lines were collected and karyotype analysis was performed as described previously [33].

### RT-PCR and quantitative RT-PCR

Total RNA samples were isolated with Trizol (Invitrogen) and cDNA was synthesized using the Superscript III First-Strand Synthesis System (Invitrogen). RT-PCR was done using specific primers with conditions as follows: 95 °C for 5 min, followed by 35 cycles at 95 °C for 30 s, 60 °C for 30 s, and 72 °C for 1 min. Gene expression levels were measured using EvaGreen dye (Biotium, Hayward, CA, USA) and a CFX96 Real-Time PCR Detection System (Bio-Rad, Hercules, CA, USA). All samples were normalized to internal controls and fold changes were calculated through relative quantification (2^−△△Ct^). Primer set information is listed in Additional file 2: Table S1.

### Alcian blue staining

Primary ZEFs and immortalized cell lines were stained with 1% Alcian blue (Millipore Sigma) in 3% glacial acetic acid solution and were visualized under a fluorescence microscope.

### Transfection and selection of primary and immortalized cells

Primary ZEFs and immortalized cell lines were transfected in serum-free medium with 1–2 μg of a *piggyBac* YFP-expressing vector and 1–2 μg of a transposase vector (CAGG PBase) using Lipofectamine 3000 reagent (Invitrogen). The transfection mixture was replaced with growth media 6 h after transfection. After culture for 2 more days, fluorescence levels were calculated using the FACSCalibur system and cell viability was measured by trypan blue staining (Millipore Sigma). To determine the most appropriate concentration for the selection of transfected cells, immortalized cells were treated with G418 at 0, 50, 100, 200, 300, 400, or 500 μg/mL, and the surviving cells were counted 1 week after treatment. Subsequently, transfected cells were treated with the selected concentration of G418 (300 μg/mL) for 1 week and the proportion of YFP-expressing cells was determined using the FACSCalibur system.

### Gene targeting vector construction

Previously described all-in-one CRISPR/Cas9 plasmids [34] were used to target the zebra finch *SOX9* gene. Guide RNA (gRNA) sequences targeting the gene were designed using Geneious Prime software, considering the on-target score. For insertion of gRNAs into the CRISPR/Cas9 plasmids (pX459), sense and antisense oligonucleotides were designed (Additional file 3: Table S2) and synthesized by Bionics (Seoul, South Korea). Annealing of sense and antisense oligonucleotides was carried out under the following thermocycling conditions: 95 °C for 30 s, 72 °C for 2 min, 37 °C for 2 min, and 25 °C for 2 min. The annealed oligonucleotides were ligated into the pX459 vector using the Golden Gate assembly method, and the constructed CRISPR/Cas9 vectors were validated by Sanger sequencing (Bionics).

### Transfection and genomic DNA sequencing analysis of the immortalized cell line

To validate the mutation efficiencies of the gRNAs, CRISPR/Cas9 plasmids containing gRNAs (2 μg) and the *piggyBac* YFP-expressing vector were co-introduced into cells using Lipofectamine 3000 reagent (Invitrogen), and then G418 selection was performed for 1 week. Genomic DNA was extracted from transfected cells and regions encompassing the CRISPR/Cas9 target sites were amplified using specific primer sets (Additional file 3: Table S2). For sequencing analysis, the PCR amplicons were annealed into the pGEM-T Easy vector and analyzed by Sanger sequencing. The sequencing results were analyzed using Geneious Prime software.

### TUNEL assay

Apoptotic cells were detected by a TUNEL assay using a TMR red in situ cell death detection kit (Roche, Basel, Switzerland), following the manufacturer’s instructions.

### Statistical analysis

Comparisons of primary and immortal cells were done via Student’s t-tests using GraphPad Prism software (GraphPad Software, La Jolla, CA, USA).

## Supplementary Information

**Additional file 1. Figure S1.** Karyotyping analysis of zebra finch primary fibroblast cells. The karyotypes of zebra finch primary fibroblast cells (passage 1) from four different embryos were analyzed. The number of karyotyped cells for each line was 20, and all karyotyped cells displayed the same karyotype.

**Additional file 2. Table S1.** Primer sequences used for RT-PCR and quantitative RT-PCR.

**Additional file 3. Table S2.** Oligonucleotide sequences used for genome editing.

## Data Availability

The datasets generated during and/or analyzed during the current study can be found in the figures, tables, and supplementary information or are available upon request.
