# Supplementary material for: Generation and characterization of genome-modified chondrocyte-like cells from the zebra finch cell line immortalized by c-MYC expression
Source: Front Zool. 2022 Jun 11;19:18. doi: 10.1186/s12983-022-00464-x (PMC9188209; doi:10.1186/s12983-022-00464-x)
Supplement: Supplementary file 1 — Additional file 1. Figure S1. Karyotyping analysis of zebra finch primary fibroblast cells. The karyotypes of zebra finch primary fibroblast cells (passage 1) from four different embryos were analyzed. The number of karyotyped cells for each line was 20, and all karyotyped cells displayed the same karyotype. [file 12983_2022_464_MOESM1_ESM.docx]

**
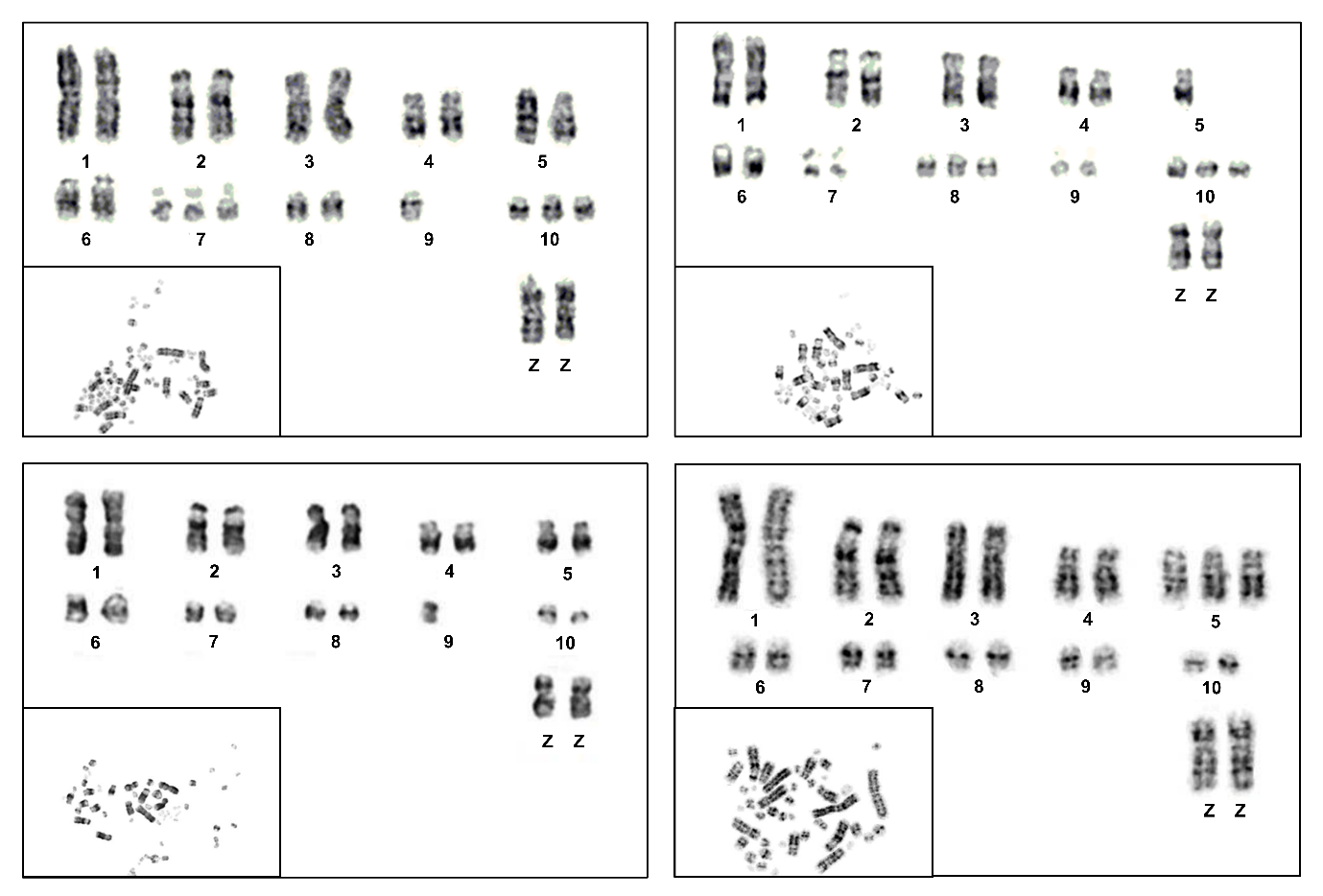
**

**Supplementary figure 1. Karyotyping analysis of zebra finch primary fibroblast cells.** The karyotypes of zebra finch primary fibroblast cells (passage 1) from four different embryos were analyzed. The number of karyotyped cells for each line was 20, and all karyotyped cells displayed the same karyotype.
