# Supplementary material for: Generation and characterization of genome-modified chondrocyte-like cells from the zebra finch cell line immortalized by c-MYC expression
Source: Front Zool. 2022 Jun 11;19:18. doi: 10.1186/s12983-022-00464-x (PMC9188209; doi:10.1186/s12983-022-00464-x)
Supplement: Supplementary file 2 — Additional file 2. Table S1. Primer sequences used for RT-PCR and quantitative RT-PCR. [file 12983_2022_464_MOESM2_ESM.docx]

**Supplementary table 1. Primer sequences used for RT-PCR and quantitative RT-PCR**

| **Usage** | **ID** | **Sequence (5’→3’)** | |
| --- | --- | --- | --- |
|  |  | **Forward** | **Reverse** |
| RT-PCR | *Ectopic Myc* | ACCCGCTCAACGACAGCAGC | ACTAGGGGCTCAGGGCTGGC |
|  | *Endo Myc* | AATCCAGCACAGAGTCCAGC | CGTCACGCAAGGCAAAGAAA |
|  | *TERT* | CAGGGGGAGGTTTGCTAAGG | GGTTCAACAACACCGCTCAC |
|  | *RB1* | CCCAGCCTAACACGGGAAAT | CCAGATTGCCAAGCGAGAGA |
|  | *ACAN* | GATCCAACCACACCGGGATT | TGCGGCACTTGTCAAAGGCT |
|  | *SOX9* | AACGCCATCTTCAAGGCGCT | GGTACTGGTCGAACTCGTTG |
|  | *SOX5* | TGAACGCCTTCATGGTGTGG | ATCTCCTCCTTGATGTGCGG |
|  | *SOX6* | ACATCAAGCGGCCGATGAAC | TCACCAATCCGCAGCTTCTT |
|  | *COL2A1* | ACCAGATCGAGAACATCCGC | CCTCAGGAAGGTCATCTGGA |
|  | *GAPDH* | CCATGCCATCACAGCCACAC | CCTTGGATGCCATGTGGACC |
| Quantitative  RT-PCR | *qRT-ACAN* | TCCGACTGATGTGTCACTGC | TCCAGAAGTTGGTTCTCCGC |
|  | *qRT-SOX9* | CATCAAGACGGAGCAGCTGA | TGTAGTAGGAGCCCGAGGAG |
|  | *qRT-SOX5* | TCCCTGACATGCACAACTCC | GTACTGCCTCATCTCCTGGC |
|  | *qRT-SOX6* | CCGACATGCACAACTCCAAC | TCACCAATCCGCAGCTTCTT |
|  | *qRT-COL2A1* | GCCCTCAACAACCAGATCGA | AAGACCTTGATGGCGTCCAG |
|  | *qRT-GAPDH* | CCACATGGCATCCAAGGAGT | AGAGCTAAGCGGTGGTGAAC |
