# Supplementary material for: Generation and characterization of genome-modified chondrocyte-like cells from the zebra finch cell line immortalized by c-MYC expression
Source: Front Zool. 2022 Jun 11;19:18. doi: 10.1186/s12983-022-00464-x (PMC9188209; doi:10.1186/s12983-022-00464-x)
Supplement: Supplementary file 3 — Additional file 3. Table S2. Oligonucleotide sequences used for genome editing. [file 12983_2022_464_MOESM3_ESM.docx]

**Supplementary table 2. Oligonucleotide sequences used for genome editing**

| **Usage** | **ID** | **Sequence (5’→3’)** |
| --- | --- | --- |
| PX459 CRISPR/Cas9  expression vectors construction | *SOX9* gRNA-1 | caccgCAAGAGAACACCTTCCCCAA |
|  |  | aaacTTGGGGAAGGTGTTCTCTTGc |
|  | *SOX9* gRNA-2 | caccgAGAGAACACCTTCCCCAAGG |
|  |  | aaacCCTTGGGGAAGGTGTTCTCTc |
|  | *SOX9* gRNA-3 | caccgCCTGAAGAAGGAGAACGACG |
|  |  | aaacCGTCGTTCTCCTTCTTCAGGc |
| Genomic DNA PCR | *SOX9*-Forward | CTCCTCGCTCCGGACTTTTT |
|  | *SOX9*-Reverse | AGGCGTTCATGGGTCTCTTG |
